# Enhancing IV Cannulation Documentation in a Resource-Limited Setting: A Quality Improvement Project From Dongola Specialized Hospital

**DOI:** 10.7759/cureus.99711

**Published:** 2025-12-20

**Authors:** Husameldin Ali Babiker, Fakher Aldeen Raft Fakher Aldeen Noman, Noha Salaheldeen Taha Mahde, Amr Elbanna Mohammed Aly, Shamat Fathi Shamat Salih, Osman Ali Osman Mustafa, Ilhan Said Jirde, Areej Ahmed Ali Mohamedalnour, Mohammed Ali Mohammed Ali, Ryan Osman Alhessen Saidahmed, Noam Mohamed Ahmed Mohamed Elbashir, Reem Sayfuldeen Hassan Othman, Marwan Abuelgasim Hamad Ibrahim, Doha Abdelbage Suliman Abdelkareem, Ahmed Adam Mohamedali Idris, Shima Ahmed, Amna Hamdelneel Ahmed Mohammed, Tsabih Emadeldin Hasbelrasoul Alata, Alaa Mohamed Elamin Osman, Mohammad Alzain Adam

**Affiliations:** 1 Emergency Medicine, Dongola Teaching Hospital, Dongola, SDN; 2 Internal Medicine, Dongola Specialized Hospital, Dongola, SDN; 3 Orthopaedic Surgery, Jalan Bani Bu Ali Hospital, Jalan Bani Bu Ali, OMN; 4 Faculty of Medicine, University of Khartoum, Khartoum, SDN; 5 Obstetrics and Gynecology, Sabt Alaiaa General Hospital, Sabt Alaiaa, SAU; 6 Emergency Medicine, Alhasaheisa Teaching Hospital, Gazira, SDN; 7 Medicine, Dongola Teaching Hospital, Dongola, SDN; 8 Pathology, Assiut University, Assiut, EGY; 9 Internal Medicine, Sudan Medical Specialization Board, Khartoum, SDN

**Keywords:** documentation audit, dongola specialized hospital, intravenous cannulation, patient safety, pdsa cycle, quality improvement, resource-limited settings

## Abstract

Background: Incomplete documentation of intravenous (IV) cannulation is a persistent challenge in hospital practice, leading to gaps in continuity of care and increased risk of complications.

Objective: This quality improvement project aimed to evaluate and enhance the completeness of IV cannula documentation at Dongola Specialized Hospital through the introduction of a standardized documentation sticker and focused staff training.

Methods: A prospective two-cycle audit was conducted between June and September 2025 using the Plan-Do-Study-Act framework. Fifty inpatient records were reviewed in each cycle from the Internal Medicine Department. Documentation was assessed against international standards for IV cannulation, including patient identifiers, insertion details, aseptic technique, site monitoring, and removal data. A structured IV cannula sticker and brief educational sessions were introduced between cycles. Data were analyzed using the chi-square test, with a significance level of p < 0.05.

Results: Baseline compliance was poor, with most parameters documented in ≤2% of records. Following the intervention, substantial improvement was observed across all fields: cannula gauge (2%→100%), indication (2%→96%), aseptic technique (2%→86%), and inserter’s name (2%→92%) (p < 0.001 for all). The documentation of removal details and Visual Infusion Phlebitis (VIP) scoring also improved markedly (p < 0.001).

Conclusion: The introduction of a structured, low-cost documentation sticker, reinforced by staff education, resulted in significant improvement in IV cannula documentation quality. This simple and sustainable intervention can strengthen patient safety and procedural accountability in resource-limited hospital settings.

## Introduction

Hydration, medication delivery, blood transfusion, and nutritional support all rely on intravenous (IV) cannulation, a crucial component of inpatient medical treatment. Its seeming simplicity, however, belies the intricacy and risk that come with incorrect insertion or neglectful maintenance. Patients are at risk for consequences such as phlebitis, infiltration, or bloodstream infection when aseptic precautions are disregarded or follow-up monitoring is inconsistent. These outcomes place a burden on healthcare resources due to longer hospital stays and higher treatment expenses, which in turn compromise patient safety [1].

Technical competence and precise documentation are equally important for safe IV cannulation procedures. The early diagnosis of infection, the timely removal of peripheral lines, and the assurance of accountability across shifts are all benefits of a correctly filled-out record of insertion and maintenance that contribute to patient safety. Thus, documentation is a clinical precaution rather than merely a bureaucratic duty. According to international authorities and national health agencies, consistent and comprehensive records are required to keep devices safe and provide continuity of care [2].

Despite this acknowledgment, numerous healthcare facilities still do not consistently document IV treatments. Important elements, including insertion site, cannula gauge, date and time, and follow-up assessment, are frequently left blank or only partially completed, according to studies. These omissions contribute to avoidable complications and decreased compliance with infection-control guidelines, creating significant gaps in patient monitoring. Furthermore, incomplete documentation makes it more difficult to assess procedural quality or assign clinical responsibility in the event of complications, thereby reducing the traceability of care [3].

Clinical documentation has been extensively used to standardize and streamline quality improvement (QI) activities in an effort to overcome these systemic deficiencies. Improvements in documentation completeness and accuracy have been observed following the implementation of standardized templates or adhesive stickers for IV cannula records, in conjunction with ongoing staff education and performance feedback. The Plan-Do-Study-Act (PDSA) cycle provides a systematic and iterative method for developing, testing, and incorporating such interventions into clinical workflows [4].

Dongola Specialized Hospital in Northern State, Sudan, implemented a targeted QI initiative to improve IV cannulation documentation after identifying recurrent deficiencies within its Internal Medicine Department. The intervention consisted of a standardized sticker-based documentation system supported by focused educational reinforcement. The aims were to improve procedural adherence, reduce IV-related complications, and establish a sustainable culture of accurate documentation. Given that enhanced documentation improves timely detection of complications and reduces risks such as phlebitis or catheter-related infections, the project was expected to positively influence patient safety outcomes. This report compares findings from the two audit cycles, before and after implementation of the intervention, to assess its effectiveness [5].

## Materials and methods

Study design and setting

This quality improvement project was conducted as a prospective, two-cycle clinical audit at Dongola Specialized Hospital, Northern State, Sudan. The hospital is a secondary-level multidisciplinary institution and a major referral center for surrounding rural areas. The high volume of IV therapy in acute care made it an appropriate setting for evaluating documentation quality and procedural safety related to IV cannulation. The audit followed the PDSA framework to ensure a systematic, iterative, and evidence-based process for identifying baseline weaknesses, implementing targeted interventions, and reassessing performance.

Study population and sampling

The study population included inpatients with documented peripheral IV cannulation during the audit period. Data were collected from the Internal Medicine Department, which has one of the highest admission rates and frequent cannula insertions. A total of 50 medical records were selected for each cycle, as this sample size offered a realistic and representative reflection of routine practice while remaining feasible within the audit’s time and resource constraints. Records were chosen using simple random sampling from the department’s admission list to minimize selection bias. Only records showing clear evidence of peripheral IV insertion were included. Cases involving central venous access, incomplete documentation, or admissions shorter than 24 hours were excluded to maintain uniformity.

Audit cycles and intervention timeline

The project was conducted over three months and divided into two audit cycles. Cycle one took place in June 2025 and assessed baseline documentation, adherence to infection control standards, and accuracy of procedural recording. The findings revealed significant gaps in completeness and consistency. A two-week intervention phase was then implemented between mid-June and early July 2025. Cycle 2 was conducted from July to September 2025 to evaluate the effectiveness and sustainability of the intervention. This timeline ensured adequate staff exposure to the new process and allowed sufficient time for post-intervention data collection.

Audit standards and outcome measures

Assessment criteria were based on internationally recognized standards, including WHO and CDC guidelines, which emphasize complete, traceable, and legible documentation for all IV procedures. Each record was evaluated for documentation of patient identifiers, insertion details, aseptic technique, indication for cannulation, and post-insertion site monitoring. Removal details, including indication, date, and staff signature, were also reviewed. The primary outcome was the overall rate of complete documentation per cycle. Secondary outcomes identified the most frequently omitted parameters to highlight areas requiring ongoing reinforcement and educational focus.

Intervention

A multifaceted intervention was implemented between the two cycles. Its central component was the introduction of a pre-printed IV cannulation documentation sticker, designed as a concise checklist attached to the patient chart with designated fields for insertion, monitoring, and removal details (Appendices). Brief educational sessions were conducted for doctors and nurses, highlighting the clinical relevance of proper documentation and demonstrating the correct application of the sticker. Posters summarizing key steps were placed across the wards as visual reminders. Supervisors and ward heads provided verbal feedback during rounds to reinforce adherence to the guidelines. The hospital administration supported the printing and integration of the sticker into IV cannulation kits, transforming documentation into a consistent and expected component of clinical care.

Data collection and statistical analysis

Data were collected using a structured Google Forms proforma to standardize entries and reduce transcription errors. Each documentation element was coded as present or absent. Data from both cycles were exported into IBM SPSS Statistics for Windows, Version 29 (Released 2022; IBM Corp., Armonk, New York). Frequencies and percentages were calculated for all parameters. Comparative analysis between cycles was performed using the chi-square test of independence, with significance set at p < 0.05. Findings were evaluated to determine the extent of improvement and to identify patterns requiring further intervention.

Ethical considerations

Ethical approval was granted by the Institutional Review Board of Dongola Specialized Hospital (Approval No: QIP-DSH-7995). No direct patient contact occurred, and all collected data were anonymized to protect confidentiality. As a quality improvement initiative aimed at enhancing routine clinical practice, the requirement for individual informed consent was waived.

## Results

In the first audit cycle, documentation of intravenous (IV) cannulation was notably incomplete across almost all parameters. Although the patient’s name was documented in all 50 records (100%), other essential identifiers and procedural details were often missing. Gender was recorded in 44 records (88%), age in all 50 (100%), and date of birth in only one (2%). Core insertion details, such as cannula gauge, indication for insertion, aseptic technique, and complications, were documented in only one file (2%) each.

Indicators of staff accountability were also largely absent. The inserter’s name appeared in just one record (2%), and both the inserter’s role and signature were documented in only one case (2%) each. The insertion site was mentioned in a single record (2%), and post-insertion site monitoring was completed in one file (2%). Documentation related to cannula removal, including date, time, and indication, was extremely limited, with each parameter recorded in only one case (2%). The remover’s name, role, and signature were similarly missing in all but one file (2% each). Observation of phlebitis using the Visual Infusion Phlebitis (VIP) score and completion of additional notes were also nearly absent, with only one documented instance (2%).

Following the introduction of the standardized IV cannula documentation sticker and short focused training, the second audit cycle showed statistically significant improvements across nearly every domain. Documentation of gender rose from 44 (88%) to 49 (98%) (χ² = 3.20; p = 0.073), and date of birth from one (2%) to 42 (84%) (χ² = 75.10; p < 0.001). Cannula gauge improved dramatically from one (2%) to full compliance in all 50 records (100%) (χ² = 88.90; p < 0.001). Similarly, the indication for insertion rose from one (2%) to 48 (96%) (χ² = 84.60; p < 0.001), and aseptic technique documentation increased from one (2%) to 43 (86%) (χ² = 78.70; p < 0.001).

Staff accountability also improved considerably. The inserter’s name was documented in 46 cases (92%), compared with only one case (2%) at baseline (χ² = 83.40; p < 0.001). Documentation of the inserter’s role and signature also improved substantially, increasing from one case (2%) to 36 cases (72%) (χ² = 69.80; p < 0.001). The site of cannulation increased from one (2%) to 44 (88%) (χ² = 80.50; p < 0.001), and post-insertion site monitoring rose from one (2%) to 32 (64%) (χ² = 60.40; p < 0.001). Removal date and time, initially recorded in only one case (2%), were documented in 30 records (60%) (χ² = 57.40; p < 0.001), while removal indication rose from one (2%) to 25 (50%) (χ² = 50.00; p < 0.001).

Documentation of the remover’s details also demonstrated strong upward trends. The remover’s name improved from one (2%) to 24 (48%) (χ² = 48.10; p < 0.001), the remover’s role from one (2%) to 19 (38%) (χ² = 35.10; p < 0.001), and the remover’s signature from one (2%) to 19 (38%) (χ² = 35.10; p < 0.001). The Visual Infusion Phlebitis (VIP) score, a critical patient safety measure, increased from one (2%) to 45 (90%) (χ² = 87.00; p < 0.001). Additional notes also showed a modest but statistically significant rise from one (2%) to seven (14%) (χ² = 6.25; p = 0.012).

Overall, mean documentation completeness increased by more than 70% between the two audit cycles, with most parameters reaching statistical significance (p < 0.001). These findings confirm that implementing a simple, structured sticker combined with staff education can significantly enhance documentation accuracy, accountability, and patient safety practices. A full comparison of documentation parameters, including corresponding chi-square and p-values, is presented in Table 1.

**Table 1:** Comparison of IV Cannula Documentation Compliance Between Audit Cycles at Dongola Specialized Hospital (n = 50 per Cycle). Frequencies are presented first, with percentages in parentheses. All statistical comparisons were performed using the chi-square test of independence (df = 1). Improvements between the two cycles were statistically significant for nearly all parameters (p < 0.001), confirming the effectiveness of the standardized IV-cannula documentation sticker and staff-training intervention.

Parameter	Cycle 1, n (%)	Cycle 2, n (%)	χ²	p-value
Patient’s name written	50 (100)	50 (100)	–	–
Patient’s gender selected	44 (88)	49 (98)	3.20	0.073
Patient’s age recorded	50 (100)	42 (84)	8.33	0.004
Date of birth recorded	1 (2)	42 (84)	75.10	<0.001
Cannula gauge selected	1 (2)	50 (100)	88.90	<0.001
Insertion indication documented	1 (2)	48 (96)	84.60	<0.001
Aseptic technique ticked	1 (2)	43 (86)	78.70	<0.001
Complications documented	1 (2)	43 (86)	78.70	<0.001
Inserter’s name written	1 (2)	46 (92)	83.40	<0.001
Inserter’s role written	1 (2)	36 (72)	69.80	<0.001
Inserter’s signature done	1 (2)	36 (72)	69.80	<0.001
Site of cannulation written	1 (2)	44 (88)	80.50	<0.001
Site monitoring ticked	1 (2)	32 (64)	60.40	<0.001
Removal date written	1 (2)	30 (60)	57.40	<0.001
Removal time recorded	1 (2)	30 (60)	57.40	<0.001
Removal indication selected	1 (2)	25 (50)	50.00	<0.001
Remover’s name documented	1 (2)	24 (48)	48.10	<0.001
Remover’s role written	1 (2)	19 (38)	35.10	<0.001
Remover’s signature done	1 (2)	19 (38)	35.10	<0.001
VIP score documented	1 (2)	45 (90)	87.00	<0.001
Additional notes written	1 (2)	7 (14)	6.25	0.012

## Discussion

IV cannula documentation at Dongola Specialized Hospital was found to be significantly improved in terms of completeness and quality after the implementation of a standardized, pre-printed documentation label and brief staff training, as shown in this quality improvement project. Compliance increased from essentially nonexistent at baseline to nearly full adherence during the re-audit cycle across all evaluated metrics. For example, there were substantial and statistically significant improvements in fields reflecting staff accountability and complication monitoring, and the proportion of records noting the insertion site, indication for insertion, and aseptic technique rose from 2% in the first cycle to over 85% in the second. Despite the operational constraints of a resource-limited setting, these outcomes demonstrate that a system-based intervention can nonetheless produce considerable gains at a low cost.

Findings from prior foreign audits are in line with the problems identified in Cycle 1. According to research by Marsh et al., there is a direct correlation between insufficient peripheral IV catheter (PIVC) documentation and increased device failure rates and infection risks [1]. Teaching hospitals also frequently report poor recording of insertion and maintenance data, which places patients at risk of preventable complications, as noted by Biswas et al. and Yagnik et al. [5,6]. The similarities between those studies and the baseline results observed in Dongola suggest that inadequate documentation of IV cannulation is a systemic issue in clinical governance and not limited to a single institution.

The improvements observed in Cycle 2 are consistent with worldwide data supporting structured quality improvement programs. Research by Cho and Kim demonstrated that focused education in conjunction with standardized documentation templates greatly improves compliance with best-practice recommendations [7]. Høvik et al. found that the PIVC-miniQ and other structured monitoring tools were useful in avoiding omissions and maintaining high compliance [8]. Stickers were an integral part of the project’s clinical workflow, serving as both visual and cognitive prompts and minimizing reliance on memory and staff-related variability. The comprehensive documentation of aseptic technique, indication for insertion, and post-intervention complications is consistent with findings from similar global initiatives and demonstrates the usefulness of such tools for hospitals facing financial limitations.

Both the Australian Clinical Care Standard [2] and the documentation structure outlined by Thompson et al. [9] support these improvements, as they emphasize the importance of thorough and accurate recordkeeping in infection prevention. Patient outcomes are directly affected by adherence to insertion and maintenance criteria, as shown in a worldwide survey by Alexandrou et al. [3]. The Dongola experience shows that, when documentation systems are streamlined and staff engagement is prioritized, these international benchmarks can be approached even in low-resource contexts.

Despite the overall success, several categories were not fully filled out, especially those related to the specifics of ongoing maintenance and removal. Despite a significant improvement from 0% to 80%, full compliance was still not achieved with flush and patency checks. There have been similar reports from other locations, where the focus is more on recording the specifics of the insertion than on the subsequent treatment. One cause of early catheter failure, as pointed out by Zingg et al., is inadequate documentation of maintenance protocols [10]. According to Bahl et al. [11], inadequate maintenance documentation was caused by heavy workloads, a lack of staff awareness, and time constraints. Continued emphasis on completing both insertion and maintenance documentation, simplification of forms, and targeted reminders may help close these gaps at Dongola.

Another important factor is the longevity of the improvement. The PDSA cycle encourages constant feedback, which helps innovative behaviors become standard workflow, as highlighted by Taylor et al. [4]. Posters, supervision, and reminders from senior staff served as a substitute for digital feedback systems used in more advanced facilities in this environment. Such feedback and audit cycles improve accountability and maintain behavior change, as shown by Henderson et al. [12]. The encouraging trend lines observed in Cycle 2 imply that comparable reinforcement, even when low-tech, can sustain improvements over time.

The advantages of structured documentation systems extend beyond improved compliance. Use of the I-DECIDED® framework resulted in decreased catheter-related problems and enhanced record completeness, as demonstrated by Ray-Barruel et al. [13]. Similarly, Hoskins et al. found that structured observational audits aid in identifying long-term gaps in practice [14]. The substantial increase in reported complications and VIP scoring suggests increased vigilance and possibly improved patient safety, even though this project did not assess clinical outcomes directly. In addition to improving communication across healthcare teams and strengthening medico-legal accountability, detailed records support clinical governance. Dongola is a busy hospital with high patient turnover and frequent staff rotations. Standardized documentation helps preserve essential information and ensures continuity of care.

This project has several limitations. First, as it was conducted at a single hospital with a relatively small sample size, the findings may have limited generalizability to other settings. Second, the short follow-up period prevents assessment of the long-term sustainability of the improvements. Future audits should therefore include multiple institutions, larger and more diverse samples, and extended monitoring periods. In addition, future studies may explore whether integrating cost-effective AI-supported documentation or monitoring tools could further improve patient safety and workflow reliability, offering a scalable option for clinical practice in resource-limited healthcare systems [15]. Linking documentation quality to clinical outcomes, such as phlebitis or catheter-related bloodstream infection rates, would provide a more comprehensive evaluation of the intervention’s impact on patient care.

In summary, this quality improvement effort demonstrates that organized, affordable, and contextually relevant interventions can substantially improve IV cannulation documentation. With staff education and visual reinforcement measures, a standardized sticker was integrated into routine workflow, resulting in near-universal compliance. This approach is practical, scalable, and effective for hospitals with limited resources seeking to improve documentation standards and patient safety culture. To keep these improvements going, we need to keep teaching, re-audit often, and add more intrusive procedures to the model.

## Conclusions

This audit demonstrated that introducing a standardized IV cannula documentation sticker, supported by brief staff training, markedly improved documentation quality at Dongola Specialized Hospital. Compliance increased from minimal baseline levels to near-complete adherence across most parameters. By ensuring more complete and accurate records, the intervention has the potential to reduce IV-related complications such as phlebitis and catheter-associated infections through earlier detection and more consistent monitoring. The approach is simple, low-cost, and easily scalable, offering practical value for other hospitals in similar resource-limited settings. Ongoing education and periodic re-audits are recommended to sustain these gains and extend the model to additional clinical procedures.

## Figures and Tables

**Figure 1:**
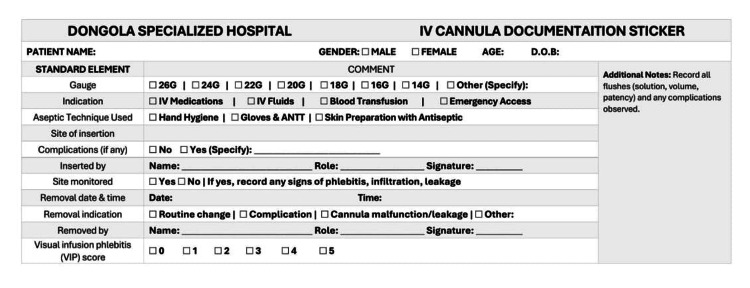
Standardized Intravenous (IV) Cannula Documentation Sticker Used in the Intervention Phase The figure shows the structured IV cannula documentation sticker implemented at Dongola Specialized Hospital during the quality improvement project. The sticker was designed to support complete and accurate documentation of IV cannulation procedures, including patient identifiers, cannula gauge, clinical indication, aseptic technique, site of insertion, complications, inserter details, ongoing site monitoring, removal data, and Visual Infusion Phlebitis (VIP) scoring. The right-hand section includes space for additional notes such as flush volume, solution type, cannula patency, and any observed complications.
